# Insights Into the Effect of *Verticillium dahliae* Defoliating-Pathotype Infection on the Content of Phenolic and Volatile Compounds Related to the Sensory Properties of Virgin Olive Oil

**DOI:** 10.3389/fpls.2019.00232

**Published:** 2019-03-05

**Authors:** Blanca B. Landa, Ana G. Pérez, Pilar Luaces, Miguel Montes-Borrego, Juan A. Navas-Cortés, Carlos Sanz

**Affiliations:** ^1^Institute for Sustainable Agriculture, Spanish National Research Council (CSIC), Córdoba, Spain; ^2^Instituto de la Grasa, Spanish National Research Council (CSIC), Seville, Spain

**Keywords:** *Verticillium dahliae*, olive oil, quality, aroma, taste, volatile, phenolics

## Abstract

Verticillium wilt, caused by the defoliating pathotype of *Verticillium dahliae*, is the most devastating soil-borne fungal disease of olive trees, and leads to low yields and high rates of tree mortality in highly susceptible cultivars. The disease is widely distributed throughout the Mediterranean olive-growing region and is one of the major limiting factors of olive oil production. Other than effects on crop yield, little is known about the effect of the disease on the content of volatile compounds and phenolics that are produced during the oil extraction process and determine virgin olive oil (VOO) quality and commercial value. Here, we aim to study the effect of Verticillium wilt of the olive tree on the content of phenolic and volatile compounds related to the sensory properties of VOO. Results showed that synthesis of six and five straight-chain carbon volatile compounds were higher and lower, respectively, in oils extracted from infected trees. Pathogen infection affected volatile compounds known to be contributors to VOO aroma: average content of one of the main positive contributors to VOO aroma, (*E*)-hex-2-enal, was 38% higher in oils extracted from infected trees, whereas average content of the main unpleasant volatile compound, pent-1-en-3-one, was almost 50% lower. In contrast, there was a clear effect of pathogen infection on the content of compounds responsible for VOO taste, where average content of the main bitterness contributor, oleuropein aglycone, was 18% lower in oil extracted from infected plants, and content of oleocanthal, the main contributor to pungency, was 26% lower. We believe this is the first evidence of the effect of Verticillium wilt infection of olive trees on volatile compounds and phenolics that are responsible of the aroma, taste, and commercial value of VOO.

## Introduction

Verticillium wilt, caused by the defoliating pathotype of *Verticillium dahliae*, is the most devastating soil-borne fungal disease of olive trees (*Olea europaea* L.). The disease is widely distributed in all Mediterranean olive-growing regions, and is one of the major limiting factors of olive oil production (Jiménez-Díaz et al., 2012). Losses from Verticillium wilt include high rates of tree mortality and reductions in fruit yield, especially in highly susceptible cultivars. Tree death tends to occur in young orchards infected by the defoliating pathotype, but adult trees are also affected, while reductions in fruit yield occur with nonlethal infections that cause drupes to shrivel, desiccate, and lose weight (Jiménez-Díaz et al., 2012). In Israel, yield reductions in irrigated “Picual” olives were estimated to be 75 and 89% at 3 and 5 years after planting, respectively (Levin et al., 2003). However, other than effects on crop yield, little is known about the effect of Verticillium wilt on oil quality obtained from infected olive trees.

The aroma and taste of virgin olive oil (VOO) are key characteristics that distinguish it from other culinary oils, and given that VOO is the only food product to undergo sensory analysis for classification (Commission Regulation No. (2568)/91,1991), these sensory properties are commercially important. Classification of VOO is regulated by the (Commission Regulation No. (2568)/91(1991) and is carried out by trained and authorized test panels that evaluate green and fruity odors, typical of healthy and freshly extracted VOO, and intensity of bitterness and pungency of the oils. Volatile and phenolic compounds responsible for the aroma and taste of VOO, respectively, are produced during the industrial oil extraction process as a consequence of destruction of cellular integrity (Romero-Segura et al., 2012; Sánchez-Ortiz et al., 2012).

Most volatile compounds present in VOO are six straight-chain carbons (C6) aldehydes and alcohols, and their corresponding esters, produced by enzymes of the lipoxygenase (LOX) pathway (Olías et al., 1993). The action of these enzymes on polyunsaturated fatty acids, such as linoleic (LA) and, especially, linolenic (LnA) acids is part of the general LOX pathway in plants that is involved in stress-induced defense responses against abiotic and biotic stresses. Biotic stress due to pathogen attack has been shown to result in the emission of these volatile compounds (Heiden et al., 2003; Prost et al., 2005; Kishimoto et al., 2008; Piesik et al., 2011; Rani et al., 2017), and their antimicrobial activity has been found to vary among compounds (Nakamura and Hatanaka, 2002; Prost et al., 2005; Shiojiri et al., 2006; Yi et al., 2009). Compounds with five straight chain carbons (C5), which are, similarly, synthesized through the LOX pathway, also affect the aroma of olive oil (Angerosa et al., 2000). The contribution of volatile compounds to VOO aroma depends on the ratio of their concentration to odor threshold, where the ratio is the odor activity value (OAV) of the volatile compound (Grosch, 1994); theoretically, the compound will only contribute to the aroma of the oil when its OAV is greater than one.

Phenolics in VOO are responsible for bitterness and pungency that are common and desirable attributes when present at low to moderate intensities (Andrewes et al., 2003; Mateos et al., 2004); however, consumers reject oils with high levels of phenolics. VOO phenolics belong to four main chemical classes: secoiridoids, lignans, flavonoids, and phenolic acids. Within these, there is a wide array of phenolic profiles that varies with cultivar, edaphic and climatic conditions, and type of industrial process used during olive oil extraction (Servili et al., 2004; Cicerale et al., 2009; García-González et al., 2010). The secoiridoid glucosides, which are especially abundant in the Oleaceae (Gariboldi et al., 1986), act as defense chemicals against herbivores and pathogens due to their antifeedant and growth inhibitory activities against insects. In olive fruit tissue, dominant secoiridoid glucosides, such as oleuropein, ligstroside, and demethyloleuropein, are substrates for phenolic synthesis through the activity of different hydrolytic and oxidative enzymes during the oil extraction process (Romero-Segura et al., 2009, 2012; García-Rodríguez et al., 2011), resulting in the formation of secoiridoid aglucones that comprise phenolic alcohol tyrosol (*p-*HPEA) and its hydroxyl derivative, hydroxytyrosol (3,4-DHPEA), and are mainly the dialdehydic forms of decarboxymethyloleuropein and decarboxymethylligstroside aglucones (3,4-DHPEA-EDA and *p-*HPEA-EDA, respectively) and aldehydic forms of oleuropein and ligstroside aglucones (3,4-DHPEA-EA and *p-*HPEA-EA, respectively) (Montedoro et al., 2002; Pérez et al., 2014). In addition to its functional properties (Lucas et al., 2011; Visioli and Bernardini, 2011; Scotece et al., 2013), *p-*HPEA-EDA (also known as oleocanthal) is understood to be the principal phenolic responsible for VOO pungency (Andrewes et al., 2003), while 3,4-DHPEA-EA drives bitterness (Mateos et al., 2004).

Verticillium wilt is one of the major limiting factors of olive oil production and other than effects on crop yield, little is known about the effect of the disease on VOO quality and commercial value. In the context of the relationship between volatile and phenolic compounds and sensory characteristics in VOO (Angerosa et al., 2000; Morales et al., 2013), we aim to study the effects of Verticillium wilt infection on these compounds as indicators of commercially important VOO quality.

## Materials and Methods

### Plant Material, Soil Inoculation Treatments and Fruit Harvesting

Three different soil types (loam, clay-loam, and sandy-loam) were selected as representative of the different soil types commonly found in Andalusia, southern Spain, the main olive growing area in the world with more that 1.5 million hectares, producing more than one-third of the world’s olive oil (Caliz et al., 2015). Since we could not repeat the experiment in different locations the use of three soils with different physicochemical properties could overcome part of this limitation. Each soil used was thoroughly mixed in a cement mixer to ensure homogeneity of each soil before use. We used a monoconidial *V. dahliae* isolate V138I from defoliated Coker 310 cotton plants from Córdoba (Spain) as a representative of the defoliating (D) pathotype in an inoculum that consisted of an infested cornmeal-sand mixture (CMS; sand:cornmeal:deionized water, 9:1:2, w/w) (Jiménez-Fernández et al., 2016). The inocuculum was thoroughly mixed with the three soil types to an inoculum rate of 3% of CMS to ensure moderate levels of disease; control soils comprised non-inoculated CMS.

For the experiment we selected the cultivar “Picual”, the most important olive cultivar in the world, representing 50% of the olives trees in Spain and therefore, approximately 20% worldwide, which is highly susceptible to the D-pathotype of *V. dahliae*. In March, we transplanted single, 1-m tall, 18-month-old “Picual” plants to 40-L pots containing one of the three types of infested or non-infested soils. Pots were buried in a field plot at the Institute of Sustainable Agriculture, Córdoba, Spain, to maintain soil temperature at a level similar to that in field soil, and plants were grown under standard drip irrigation and fertilization conditions. There were a total of 50 and 25 replicates of inoculated and non-inoculated plants, respectively, which were arranged in a completely randomized block design in five blocks within the experimental plot (*i.e.*, 10 and five inoculated and non-inoculated plants, respectively, per block). Disease incidence in plants was assessed monthly, as the proportion of plants showing disease symptoms, and severity of foliar symptoms was assessed using a 0 to 4 rating scale, according to the proportion of affected leaves and twigs (Jiménez-Fernández et al., 2016).

All the olive fruits per tree were manually harvested in November of the second year and weighed. Then, in each experimental block fruits of the same treatment (soil type and inoculation treatment) with similar ripening index (RI = 2) were combined to form a minimum of 2 kg of olive fruits per treatment and block. For this purpose, the fruits with total green color and reddish brown or black skin were discarded, so that most of the fruits had green skin with reddish spots, corresponding to RI = 2 according to García and Yousfi (2005).

### Oil Extraction

Virgin olive oil was extracted using an Abencor lab plant (Comercial Abengoa, S.A., Seville, Spain) that mimics the industrial process of VOO production (Martínez et al., 1975). Fruit were milled at 3000 rpm using a stainless steel hammer mill equipped with a 5-mm sieve, followed by a malaxation step at 28°C for 30 min. Then, the malaxed olive paste was centrifuged in a basket centrifuge for 1 min at 3500 rpm, and the oil was decanted, paper filtered, and stored at −20°C under nitrogen atmosphere prior to analysis.

### Phenolic Compound Analysis

Phenolic compounds were isolated from the oils using solid phase extraction, as described by Mateos et al. (2001). A solution of *p*-hydroxyphenyl-acetic acid (46.4 μg mL^−1^) and *o*-coumaric acid (9.6 μg mL^−1^) in methanol was used as internal standard, which was added (0.5 mL) to each oil sample (2.5 g) before the extraction procedure. Methanol was evaporated in a rotary vacuum evaporator at 40°C and the residue was dissolved in 6 mL of hexane. A diol-bonded phase cartridge (Supelco, Bellefonte, PA, United States) was conditioned by passing first 6 mL of methanol and then 6 mL of hexane. The oil solution was loaded onto the column and the solvent was pulled through. Then, the phase was washed twice with 3 mL of hexane and 4 mL of hexane/ethyl acetate (90:10, v/v). Finally, the phenolics were eluted from the cartridge with 10 mL of methanol, and the solvent was evaporated in a rotary vacuum evaporator at 40°C. The residue was dissolved in 500 μL of methanol/water (1:1, v/v), which constituted the VOO phenolics extract. Phenolics extracts were analyzed using a Beckman Coulter liquid chromatographic system equipped with a diode array detector (System Gold 168) and a Mediterranea Sea 18 column (internal diameter: 4.0 × 250 mm; particle size: 5 μm) (Teknokroma, Barcelona, Spain) (after Luaces et al., 2007). Elution was performed at a flow rate of 1.0 mL min^−1^, using water/phosphoric acid (99.5:0.5) (solvent A) and methanol/acetonitrile (50:50) (solvent B) as the mobile phases and the following elution program: (A) 0–25 min, 5–30% solvent B; (B) 25–35 min, 30–38% solvent B; 35–40 min, 38% solvent B; 40–45 min, 38–100% solvent B. Two phenolic extracts were isolated from each oil sample and duplicate analyses were carried out for each phenolic extract. Quantification of phenols and lignans was done at 280 nm except for ferulic acid, while flavones and ferulic acid quantification was carried out at 335 nm. Response factors (Rf) were used for each phenolic compound as stated by Mateos et al. (2001) [concentration (μg g^−1^) = Rf × area compound/area internal standard]. Compounds were identified according to their UV–Vis spectra, and confirmed using HPLC/ESI-qTOF-HRMS with available standards on a Dionex Ultimate 3000 RS U-HPLC liquid chromatograph system (Thermo Fisher Scientific, Waltham, MA, United States), equipped with a 3-μm particle size Mediterranea Sea 18 column and operated for mass analysis using a micrOTOF-QII High Resolution Time-of-Flight mass spectrometer with qQ-TOF geometry (Bruker Daltonics, Bremen, Germany) equipped with an electrospray ionization interface. Mass spectra were obtained in MS full scan mode. Data were processed using TargetAnalysis 1.2 software (Bruker Daltonics, Bremen, Germany).

The extracted phenolic compounds were grouped according to the phenolic alcohol they contained (tyrosol or hydroxytyrosol), lignans, flavonoids, and phenolic acids (Table 1).

**Table 1:** Volatile and phenolic compounds analyzed in the olive oils obtained from olive trees cv. Picual infected (Vd) and non-infected (control) by D-*Verticillium dahliae*.

Class of compound	Compound	Code	ID^*a*^	RI^*b*^	Rt (min)
	VOLATILES				
C6/LnA	(E)-hex-3-enal	6C-1	MS, RI, Std	1149	21.9
	(Z)-hex-3-enal	6C-2	MS, RI, Std	1158	22.3
	(Z)-hex-2-enal	6C-3	MS, RI, Std	1209	26.1
	(E)-hex-2-enal	6C-4	MS, RI, Std	1226	27.2
	(E)-hex-3-enol	6C-5	MS, RI, Std	1366	37.0
	(Z)-hex-3-enol	6C-6	MS, RI, Std	1386	38.3
	(E)-hex-2-enol	6C-7	MS, RI, Std	1407	39.8
C6/LA	hexanal	6C-8	MS, RI, Std	1088	18.2
	hexan-1-ol	6C-9	MS, RI, Std	1355	36.3
C5/LnA	pent-1-en-3-one	5C-1	MS, RI, Std	1025	14.7
	(Z)-pent-2-enal	5C-2	MS, RI, Std	1113	19.8
	(E)-pent-2-enal	5C-3	MS, RI, Std	1135	21.2
	pent-1-en-3-ol	5C-4	MS, RI, Std	1163	23.2
	(Z)-pent-2-en-1-ol	5C-5	MS, RI, Std	1324	34.1
	(E)-pent-2-en-1-ol	5C-6	MS, RI, Std	1316	33.6
	pentene dimer - 1	5C-7	MS, RI	959	11.7
	pentene dimer - 2	5C-8	MS, RI	967	12.0
	pentene dimer - 3	5C-9	MS, RI	1012	14.0
	pentene dimer - 4	5C-10	MS, RI	1026	14.9
	pentene dimer - 5	5C-11	MS, RI	1078	17.9
	pentene dimer - 6	5C-12	MS, RI	1087	18.4
	pentene dimer - 7	5C-13	MS, RI	1090	18.5
C5/LA	pentan-3-one	5C-14	MS, RI, Std	980	12.4
	pentanal	5C-15	MS, RI, Std	983	12.6
	pentan-1-ol	5C-16	MS, RI, Std	1253	29.3
Esters	hexyl acetate	E-1	MS, RI, Std	1278	31.0
	(E)-hex-2-en-1-yl acetate	E-2	MS, RI, Std	1341	35.1
	(Z)-hex-3-en-1-yl acetate	E-3	MS, RI, Std	1323	34.0
	methyl acetate	E-4	MS, RI, Std	829	7.5
	ethyl acetate	E-5	MS, RI, Std	891	9.0
	methyl hexanoate	E-6	MS, RI, Std	1193	25.0
	ethyl hexanoate	E-7	MS, RI, Std	1232	28.3
BC	2-methyl-butanal	BC-1	MS, RI, Std	915	9.9
	3-methyl-butanal	BC-2	MS, RI, Std	919	10.0
	2-methyl-butan-1-ol	BC-3	MS, RI, Std	1211	26.4
Terpene	limonene	T-1	MS, RI, Std	1201	26.0
	PHENOLICS				
HTyr derivatives	hydroxytyrosol	3,4-DHPEA	MS, Std		8.9
	hydroxytyrosol acetate	3,4-DHPEA acetate	MS		23.9
	decarboxymethyloleuropein	3,4-DHPEA-EDA	MS		30.4
	aglucone – dialdehyde				
	oleuropein aglucone – aldehyde	3,4-DHPEA-EA	MS		42.5
Tyr derivatives	tyrosol	p-HPEA	MS, Std		12.6
	decarboxymethylligstroside	p-HPEA-EDA	MS		35.8
	aglucone – dialdehyde				
	ligstroside aglucone – aldehyde	p-HPEA-EA	MS		49.0
Lignans	pinoresinol		MS, Std		37.3
	1-acetoxypinoresinol		MS, Std		37.5
Flavonoids	luteolin		MS, Std		39.5
	apigenin		MS, Std		46.0
Phenolic acids	vanillic acid		MS, Std		16.2
	*p*-coumaric acid		MS, Std		22.0
	cinnamic acid		MS, Std		37.4
	ferulic acid		MS, Std		24.2

*^a^Compounds were identified according to the following: MS, mass spectrum matching Wiley/NBS and NIST libraries; RI, retention index in agreement with literature; Std, coelution with chemical standard. ^b^Kovats indeces obtained by Pérez et al. (2016) and agreement with the Flavornet database http://www.flavornet.org/.*

### Volatile Compound Analysis

We analyzed volatile compounds according to Pérez et al. (2016) using headspace solid-phase microextraction (SPME) and gas chromatography (GC). Oil samples (0.5 g) were taken in 10 mL vials and placed in a vial heater at 40°C. After 10 min of equilibrium time, volatile compounds from headspace were adsorbed on a SPME fiber DVB/Carboxen/PDMS 50/30 μm (Supelco Co., Bellefonte, PA, United States). Sampling time was 50 min at 40°C. Desorption of volatile compounds trapped in the SPME fiber was done directly into the GC injector. Compounds were identified on a 7820A/GC-5975/MSD system (Agilent Technologies, Santa Clara, CA, United States), housing a DB-Wax capillary column (internal diameter: 60 × 0.25 mm; film thickness: 0.25 μm; J&W Scientific, Folsom, CA, United States) under the following conditions: injection port operated in splitless mode at 250°C; He carrier gas flow rate of 1 mL min^−1^; column held for 6 min at 40°C, then programmed at 2°C min^−1^ to 168°C; the mass detector was operated in electronic impact mode at 70 eV; source temperature set at 230°C; and, the mass spectra scanned at 2.86 scans s^−1^ in the m/z 40–550 amu range. Compounds were identified by mass spectrum comparison with Wiley/NBS and NIST libraries, retention index in agreement with the literature, and co-elution with chemical standards when available. For quantification, the volatile fraction was analyzed three times on a HP-6890 GC system (Agilent Technologies, Santa Clara, CA, United States) equipped with the same column as previously, and operated under the following operating conditions: N_2_ as the carrier gas at 17 psi; injector and detector at 250°C; oven held for 6 min at 40°C and then programmed at 2°C min^−1^ increase to 168°C. Calibration curves were obtained using re-deodorized, high-oleic sunflower oil containing pure standards. The concentration ranges and the limits of quantification were established according to the typical volatile contents in VOO. Linear regression curves were found for all compounds, with regression coefficients greater than 0.99, and calculated Rf used for quantitation [concentration (ng/g) = Rf × area compound]. During the sample analysis, a blank that did not contain oil and a mixture of volatile standards were repeatedly run.

Extracted volatile compounds were clustered into groups according to the number of carbons in the straight-chain molecules (C6 or C5) and the polyunsaturated fatty acid of origin (LnA or LA), esters, branched-chain (BC) molecules, and terpene (Table 1).

### Statistical Analyses

An initial, non-supervised multivariate hierarchical clustering analysis, using Pearson’s correlation to measure distance and an average Ward clustering algorithm of all the volatile and phenolic compounds from the different treatments was performed using MetaboAnalyst 4.0^1^ (Chong et al., 2018), and run combining both groups or independently. Then, a supervised clustering method, principal least square-discriminant analysis (PLS-DA), of either all volatile and phenolic compounds or only those volatile compounds with a role in oil aroma plus *p-*HPEA-EDA and 3,4- DHPEA-EA, was used to reduce the number of variables (metabolites) in high-dimensional metabolomics data to produce robust and easy-to-interpret models (Chong et al., 2018). Additionally, data on disease development, fruit yield, and metabolite quantification were subjected to standard analysis of variance (ANOVA) with the GLM procedure in SAS. Data were tested for normality by the Shapiro-Wilk test and transformed if needed to obtain normal distributions. All analysis followed a factorial design in a randomized complete block design; the factors were inoculation treatment (*V. dahliae*, control), soil type (loam, clay-loam, and sandy-loam), and the interaction between those factors. When significant differences were found mean comparisons among soil types were performed using Tukey HSD test (*P* = 0.05).

## Results and Discussion

### Verticillim Wilt Development

Moderate to low disease symptoms, characterized as early dropping of symptomless, green leaves, and necrosis and death of some main branches, started to develop in plants inoculated with the D-*V. dahliae* V138I isolate two months after inoculation. Symptom severity increased during the growing season, reaching a maximum in early summer. Control plants did not develop Verticillium wilt symptoms during the experiment. Maximum disease severity was observed in July, and was greater in plants grown in sandy-loam soil (severity score: 1.9) than in clay-loam (severity score: 0.92) and loam (severity score: 0.38) soils (*P* = 0.002) (Table 2 and Supplementary Table 1). Disease incidence and mortality in inoculated soils was significantly higher (*P* < 0.017) for plants grown in the sandy-loam soil (Table 2 and Supplementary Table 1). Incidence of Verticillium wilt in olive and prevalence of the D-pathotype in southern Spain has been reported to be lower in clay soils than in sandy, loam, clay-loam, and sandy-loam soils (Jiménez-Díaz et al., 2012). We found that Verticillium wilt infection decreased (*P* = 0.008) olive yield, but there was no effect (*P* > 0.147) of type of soil or type of soil × treatment interaction: mean yield in the control was 458.7 ± 35.6 g tree^−1^, whereas in infected trees was 344.1 ± 28.5 g tree^−1^, representing a net reduction in yield of about 25% (Table 2 and Supplementary Table 1). We also found there were more trees with zero yield in the *V. dahliae* treatment (32.0%) than in the control (9.3%), although this effect varied depending on the soil type (*P* < 0.001; Table 2 and Supplementary Table 1). Previous studies in Mediterranean olive orchards with 1 to 4.5% Verticillium wilt incidence estimated annual harvest losses of 1 to 3% (Thanassoulopoulos et al., 1979; Al-Ahmad and Mosli, 1993), and the impact of D-*V. dahliae* infection on yield reduction in irrigated “Picual” olive trees at 3 and 5 years after planting has been estimated to be 87 and 73%, respectively (Levin et al., 2003).

**Table 2:** Verticillium wilt development and yield of olive trees cv. “Picual” grown in three types of soil infected and non-infected^*a*^ by D-*Verticillium dahliae*.^∗^

Variable	Soil type	*V. dahliae* inoculated plants^*b*^	Control plants^*b,c*^
Death plants (%)	Clay-loam	18.0 A	0.0
	Loam	4.0 AB	0.0
	Sandy-loam	28.0 B	0.0
Disease Incidence (%)	Clay-loam	26.0 A	0.0
	Loam	24.0 A	0.0
	Sandy-loam	30.0 B	0.0
Mean Disease Severity (0–4 escale)	Clay-loam	0.92 A	0.0
	Loam	0.38 A	0.0
	Sandy-loam	1.92 B	0.0
Trees with no yield (%)	Clay-loam	30.0 A	12.0 A
	Loam	14.0 A	8.0 A
	Sandy-loam	52.0 B	8.0 A^∗^
Mean yield per tree (g)	Clay-loam	368.4	427.2^∗^
	Loam	417.3	452.6^∗^
	Sandy-loam	246.6	459.4^∗^

*^a^For each soil type data were obtained from a total of 50 and 25 replicates of inoculated and non-inoculated plants, respectively, which were arranged in a completely randomized block design in five blocks within the experimental plot. ^b^Means in column within each inoculation treatment followed by different letters indicate that they are significantly different (P < 0.05) among soil types, according to Tukey HSD test (See Supplementary Table 1 for ANOVA results). Only significant differences are shown. ^c^Means followed by an asterisk indicate that are significantly higher (P < 0.05) than those in the other inoculation treatment within each soil type (see Supplementary Table 1 for ANOVA results).*

### Effect of *Verticillium dahliae* Infection on the Volatile Profiles of VOO

**FIGURE 1:**
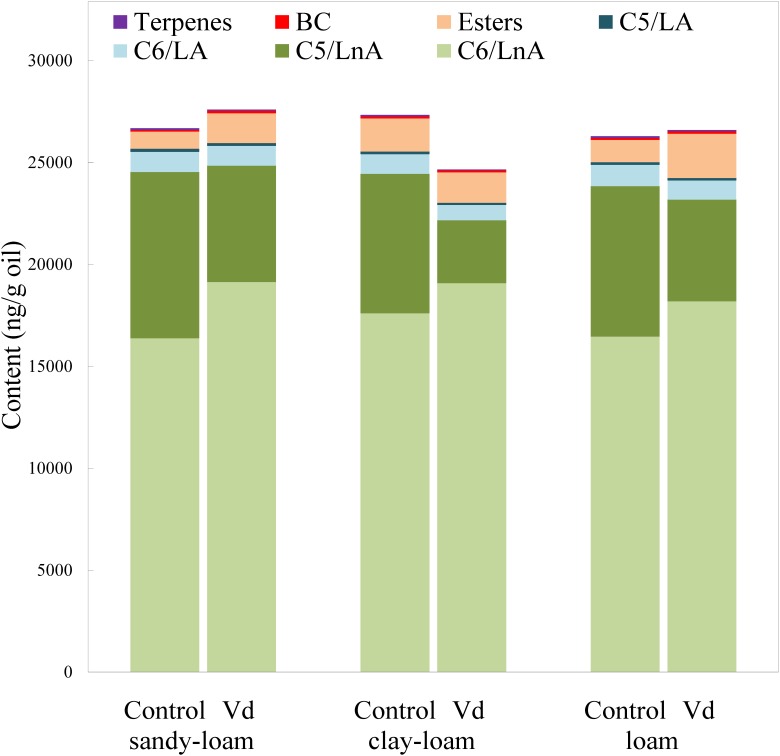
Content (ng g^−1^ oil) of the main groups of volatile compounds in oils extracted from olive trees grown in three different soils (sandy-loam, clay-loam, and loam) infected (Vd) and non-infected (control) by D-*Verticillium dahliae*.

In addition to assessing impacts of *V. dahliae* infection on yield, we analyzed its effect on volatile and phenolic fractions that are related to the sensory quality of the VOO. In general, *V. dahliae* inoculation affected significantly (*P* < 0.05) the content of several volatile compounds, whereas the effect of soil type was less evident (Supplementary Table 2). While the LOX pathway is involved in plant signaling of activation of stress-induced defense responses, the products of the LOX pathway, especially the hexenals, may directly repel pests and pathogens (Deng et al., 1993; Hildebrand et al., 1993; Vaughn and Gardner, 1993; Bate and Rothstein, 1998; Rani et al., 2017). We found that the main group of volatile compounds in the oils comprised six straight-chain carbon compounds (C6/LnA), derived from LnA (Figure 1), and produced through the LOX pathway (Olías et al., 1993). *V. dahliae* infection by the D-pathotype induced a significant increase (*P* = 0.02; Supplementary Table 2) in the average content of C6/LnA compounds independently of soil type, from 63 to 72% of total volatiles. These results support previous studies on infection by other pathogenic microorganisms in different plant species (Heiden et al., 2003; Prost et al., 2005; Kishimoto et al., 2008; Piesik et al., 2011; Rani et al., 2017). We found that the C6/LnA aldehyde group was significantly (*P* = 0.002) affected by infection, with the dominant (*E*)-hex-2-enal (6C-4) being significantly increased (*P* = 0.001) in D-*V. dahliae* inoculated plants (Supplementary Table 2), averaging 66% of C6/LnA content in oils from the control and 80% from infected trees. This compound is one of the main contributors to VOO aroma, due to its high level of content that is typical in oils from olive species (Luna et al., 2006; Pérez et al., 2016), and its relatively low odor threshold (424 ng g^−1^ oil; Reiners and Grosch, 1998).

Unlike the C6/LnA group of compounds, D-*V. dahliae* infection caused a significant decrease (*P* < 0.001) in content of C5/LnA compounds that was from 28 to 17% of total volatiles, and affected the content of all types of compounds within this group almost equally. On average, the C5/LnA group of compounds mostly comprised pentene dimers; however, they do not appear to have any role in VOO aroma according to their odor thresholds (Pérez et al., 2016). Coversely, the compound pent-1-en-3-one (5C-1) that has been shown to be an undesirable contributor to VOO aroma, as it creates a green-pungency to oil aroma (Angerosa et al., 2000), was significantly lower (*P* < 0.001), about 50%, in D-*V. dahliae* infected trees, which could affect the sensory quality of VOO given its high OAV due to its extremely low detection threshold (0.73 ng g^−1^ oil; Reiners and Grosch, 1998). In contrast, the contribution with desirable fruity green notes to the aroma of pent-2-en-1-ols (5C-5 and 5C-6) is reduced in oils from infected plants.

Oil content of esters synthesized through the LOX pathway (LOX esters) is commercially important because they are the main contributors to oil fruitiness that are evaluated in the regulated sensory analysis for commercial grading. Among them, (*Z*)-hex-3-en-1-yl acetate (E3) may be the most important due to its higher content and lower odor threshold than other esters, such as hexyl acetate (E1) and (*E*)-hex-2-en-1-yl acetate (E2) (Pérez et al., 2016). From those compounds, only the E1 and E2 esters showed significant higher (*P* = 0.001) values in D-*V. dahliae* inoculated plants as compared to control plants (Supplementary Table 2).

**FIGURE 2:**
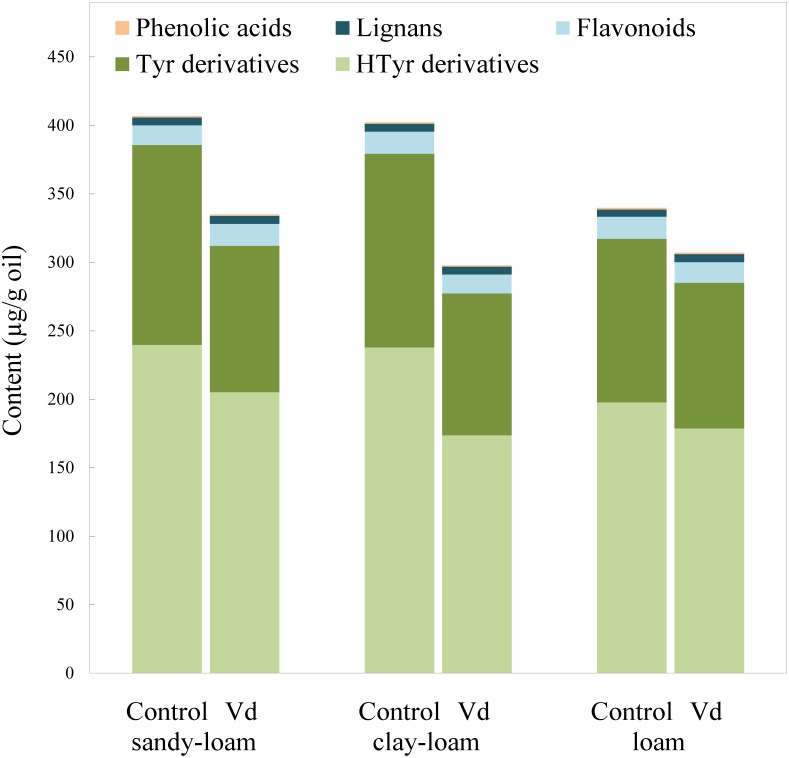
Content (μg g^−1^ oil) of the main groups of phenolic compounds in oils extracted from olive trees grown in three different soils (sandy-loam, clay-loam, and loam) infected (Vd) and non-infected (control) by D-*Verticillium dahliae*.

Although low concentrations of BC volatile compounds were found in the oils (Figure 2 and Supplementary Table 2), it is possible that they play a key role in oil aroma, because BC aldehydes 2 and 3-methyl-butanal are significant contributors to VOO aroma (Aparicio and Morales, 1995) due to their low odor thresholds (5 ng g^−1^ oil; Reiners and Grosch, 1998) that provide OAV values >1. In contrast, we found that content of 2-methyl-butan-1-ol (BC-3) was below its odor threshold (420 ng g^−1^ oil; Aparicio and Luna, 2002), indicating it did not contribute to oil aroma. This compound is associated with unpleasant fusty notes in the aroma of VOO (Angerosa et al., 1996). We found that terpenes were present in the volatile fraction of VOO, albeit at low concentrations. Although limonene was the dominant terpene, it was not an important contributor to the aroma of VOO, because its OAV was <1, although showed significant lower (*P* = 0.001) values in inoculated plants (Supplementary Table 2).

### Effect of *Verticillium dahliae* Infection on the VOO Phenolic Profiles

There are four main groups of olive oil phenolics, comprising compounds that contain tyrosol (*p-*HPEA) or its hydroxyl derivative hydroxytyrosol (3,4-DHPEA) in their molecules, lignans, flavonoids, and an array of simple phenolic acids (Figure 2). Our finding that tyrosol and hydroxytyrosol derivatives were the most abundant phenolics in the oils, especially those with a secoiridoid chemical structure, such as the aldehydic forms 3,4-DHPEA-EA and *p-*HPEA-EA, and the dialdehydic forms 3,4-DHPEA-EDA and *p-*HPEA-EDA, supports previous findings for the olive cultivar (“Picual”) used in the study (García-Rodríguez et al., 2017). On average, the most abundant were secoiridoids derived from hydroxytyrosol, 3,4-DHPEA-EDA, and 3,4-DHPEA-EA. In general, we found that infection of olive trees with D-*V. dahliae* led to oils with a significant (*P* = 0.007) lower content of phenolic compounds (Figure 2 and Supplementary Table 2), as a result of reductions in content of tyrosol and hydroxytyrosol derivatives, whereas the soil type only have an influence on 3,4-DHPEA and 3,4-DHPEA acetate (Supplementary Table 2). Unlike the content of the C6 volatiles, average content of the tyrosol derivatives in oil from infected trees was 22% lower (*P* = 0.007) than those from the non-infected control trees, and content of the hydroxytyrosol derivatives 17% lower. In contrast, the remaining main phenolic groups in the oils (flavonoids and lignans) were unaffected by D-*V. dahliae* tree infection (Figure 2 and Supplementary Table 2). Thus, there were contrasting effects of D-*V. dahliae* infection on the content of volatile compounds and phenolics. In young *Arabidopsis* seedlings, Bate and Rothstein (1998) demonstrated that 2-hexenal induces several genes known to be involved in the defense response of the plant, such as those related to the phenylpropanoids pathway that leads to the production of anthocyanins. This greater use of phenylalanine in the synthesis of phenylpropanoids and anthocyanins may explain a lower availability for the synthesis of secoiridoid compounds that are produced, in part, from the hydroxylated derivatives of phenylalanine tyrosine and dihydroxyphenylalanine (Saimaru and Orihara, 2010).

Among the oil phenolics, *p-*HPEA-EDA and 3,4-DHPEA-EA have been shown drive the taste properties of VOO. We found that average content of *p-*HPEA-EDA (oleocanthal), which is likely to be the main phenolic responsible for VOO pungency (Andrewes et al., 2003), was 25% lower (*P* = 0.009) in the oils from D-*V. dahliae* infected trees, and it is likely there would be an associated lowered perception of pungency in these oils. Similarly, content of 3,4-DHPEA-EA, which may be a key compound for bitterness in VOO (Mateos et al., 2004), was almost 20% lower (*P* = 0.018) in oils of infected trees. Mateos et al. (2004) developed an equation that includes oil content of 3,4-DHPEA-EA to calculate VOO bitterness; according to this calculation, we estimated that average perception of bitterness was around 17% lower in oils obtained from infected trees.

### Differences of Oils From Infected Plants

**FIGURE 3:**
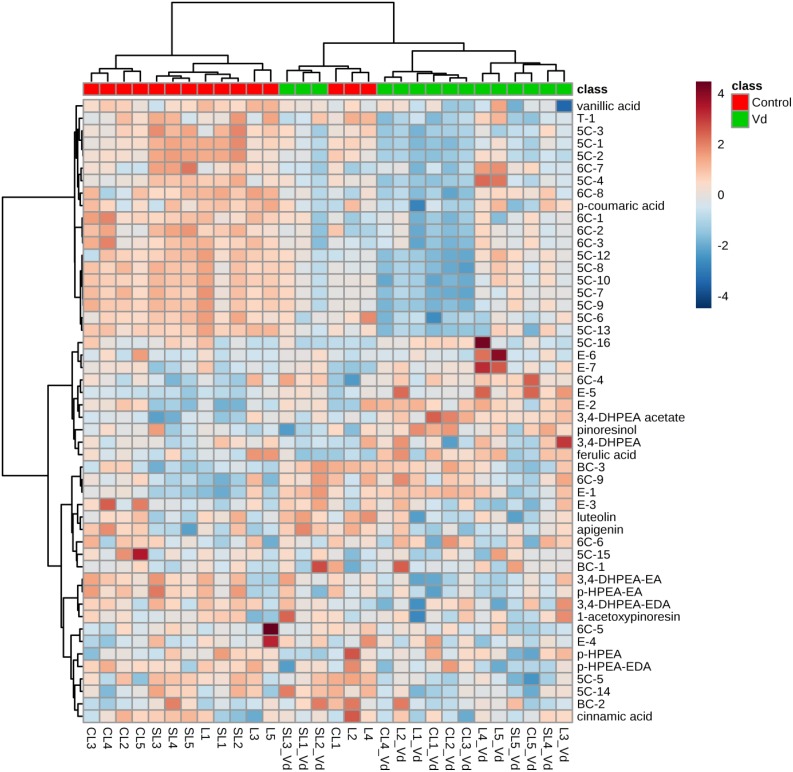
Hierarchical cluster analysis and heatmap of quantified volatile and phenolic compounds in the oils extracted from olive trees grown in three different soils (L: loam; CL: clay-loam; and, SL: sandy-loam) infected (Vd) and non-infected (control) by D-*Verticillium dahliae*. Numbers correspond to the VOO extracted from trees grown in each of five blocks in the experimental plot.

In general, hierarchical clustering analysis using all volatile and phenolic compounds differentiated oils obtained from infected olive trees from those obtained from non-infected control trees (Figure 3). There was a trend to group the oils from non-infected trees by soil type, where oil from plants grown in the sandy-loam (SL) formed a distinct cluster to oils obtained from those grown in loam (L) and clay-loam (CL) soils (Figure 3). This general trend of differentiating the VOO obtained from Verticillium wilt infected or non-infected trees was kept when hierarchical clustering analysis was run only with the volatile compounds or with the phenolic compounds, with only a few exceptions (Supplementary Figures 1A,B).

**FIGURE 4:**
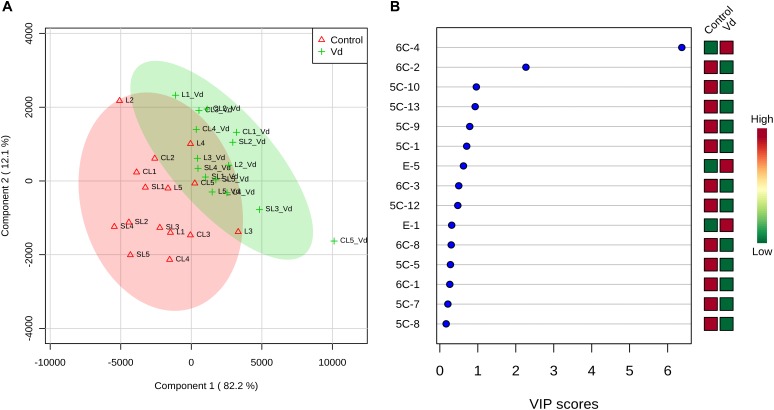
**(A)** Partial least square-discriminant analysis (PLS-DA) 2D score plot of oils extracted from olive trees grown in three different soils (L: loam; CL: clay-loam; and, SL: sandy-loam) infected (Vd) and non-infected (control) by D-*Verticillium dahliae*. Red: control; Green: *V. dahliae* inoculated trees. The model was established using three principal components; explained variance is in parentheses. **(B)** Loading importance of metabolites in the first PLS-DA component. Colored boxes (red: control; green: *V. dahliae* inoculated trees) indicate relative concentrations of the corresponding metabolite in each group.

PLS-DA of all volatile and phenolic compounds showed a separation of the VOO obtained from infected and non-infected trees, indicating there was an altered state of metabolite levels in the VOO, as a result of tree health (*P* < 0.01; Figure 4A). PLS-DA ranked the compounds using variable importance in projection (VIP) scores at *P* ≤ 0.05 with a *post-hoc* analysis (Fisher’s LSD) (Figure 4B), and showed that, as expected, (*E*)-hex-2-enal (6C-4) had the highest VIP score, showing values were higher for oils obtained from D-*V. dahliae* infected trees. In contrast, 1-penten-3-one (5C-1) had a moderate VIP value, where content was higher in oils obtained from non-infected control plants.

**FIGURE 5:**
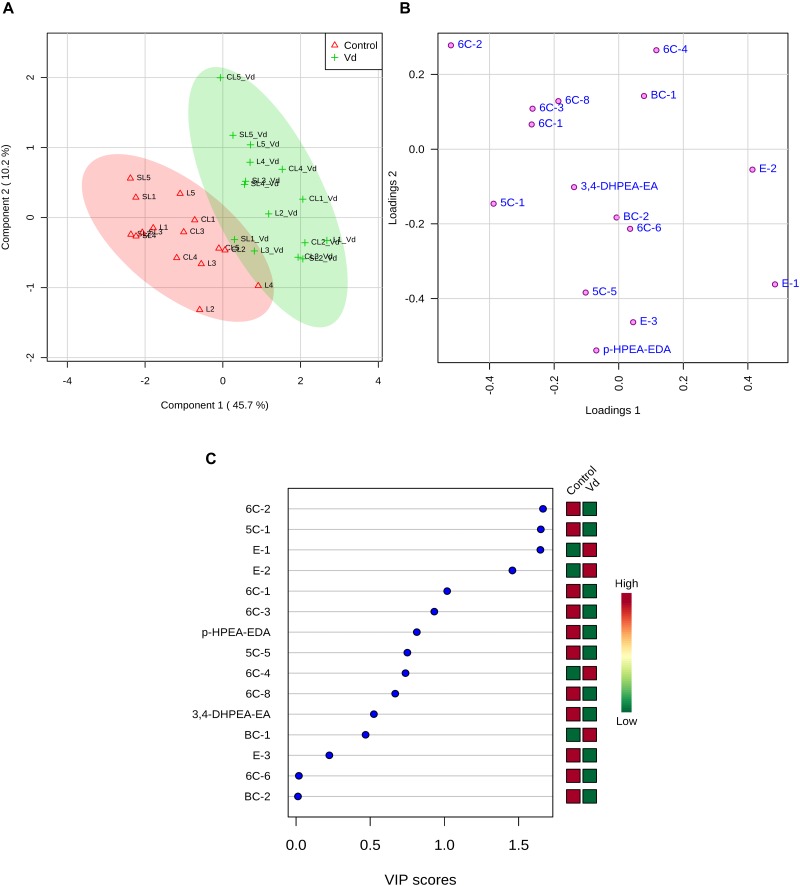
**(A)** Sparse partial least square-discriminant analysis (PLS-DA) 2D score plot of volatile compounds with odor active values (OAV) > 1 and *p-*HPEA-EDA and 3,4- DHPEA-EA from oils extracted from olive trees grown in three different soils (L: loam; CL: clay-loam; and, SL: sandy-loam) infected (Vd) and non-infected (control) by D-*Verticillium dahliae*. Red: control; green: *V. dahliae* inoculated trees. The model was established using three principal components and explained variance is shown in parentheses. **(B)** Loadings between the selected PCs. **(C)** Loading importances of metabolites in the first PLS-DA component. Colored boxes (red: control; green: *V. dahliae* inoculated trees) indicate relative concentrations of the corresponding metabolite in each group.

The contribution of each volatile compound to the aroma of VOO is dependent on its concentration and odor threshold. We found that only a few volatile compounds were present at levels where they contributed to oil aroma (OAV > 1) (Supplementary Table 3). We used PLS-DA to analyze volatile compounds with OAV > 1 and *p-*HPEA-EDA and 3,4-DHPEA-EA as compounds responsible for the pungent and bitter taste notes of the oils (Figure 5). The selected volatile compounds are desirable for VOO aroma, except pent-1-en-3-one (5C-1) that creates unpleasant sensory profiles, according to literature (Aparicio and Morales, 1995; Angerosa et al., 2000). PLS-DA analysis showed a clear separation of VOO obtained from D-*V. dahliae* infected and non-infected trees (Figure 5A). We assessed the accuracy of our predictive model using the leave-one-out cross-validation method and randomized permutations, and found that there was discrimination between VOO obtained from D-*V. dahliae* infected and non-infected trees (*P* < 0.001), where components 1 and 2 explained 56% of the variation in content of compounds responsible for oil flavor notes, supporting the observed results from the analysis of all compounds (Figure 4A). The loadings plot (Figure 5B) clearly separates desirable and undesirable compounds: vectors for phenols responsible for the bitter and pungent notes (3,4- DHPEA-EA and *p-*HPEA-EDA) and the only non-desirable volatile compound (pent-1-en-3-one, 5C-1) are located in the plot quadrants that correspond to the negative component 1 axis. In contrast, the area with positive component 1 is, according to the literature, more related to pleasant flavor notes and contains (*E*)-hex-2-enal (6C4) as well as some of the esters responsible for the fruity odor notes (E-1, E-2, E-3) and 3-methyl-butanal (BC-1). Oils obtained from trees infected with D-*V. dahliae* are located along the positive component 1 axis in Figure 5A, indicating they create more desirable flavor than the oils from non-infected trees. Changes in essential oil composition due to pathogen infection have been reported in some *Ocimum basilicum* (sweet basil) and *Mentha* (mint) spp. (Nagai et al., 2011; Smitha and Rana, 2015). PLS-DA ranked 15 metabolites by VIP score (Figure 5C), and showed the only non-desirable volatile compound, pent-1-en-3-one (5C-1), had the highest VIP score and was discriminated by treatment, while the main compounds responsible for aroma, (*E*)-hex-2-enal (6C4) and (*Z*)-hex-3-en-1-yl acetate (E-3), and the two phenolics (3,4- DHPEA-EA and *p-*HPEA-EDA) had lower, although moderate, VIP values.

## Conclusion

We found that infection of olive plants by the defoliating pathotype of *V. dahliae* apart from reducing olive fruit yield promoted an increase in the synthesis of C6 volatile compounds and a decrease in C5 compounds that are the main contributors to VOO aroma. Plant infection led to a reduction in the synthesis of the main phenolic compounds responsible for the taste sensory characteristics of VOO. There was a separation of oils from D-*V. dahliae* infected and non-infected trees, especially when considering the metabolites that really contribute to VOO flavor. To our knowledge, this is the first demonstration of the effect of Verticillium wilt infection on volatile compounds and phenolics responsible for VOO flavor. Future work integrating changes in plant metabolites combined with sensory evaluation are necessary to decipher the plant response to *V. dahliae* infection and the sensory quality of VOO.

## Data Availability

The datasets generated for this study are available on request to the corresponding author.

## Author Contributions

BBL and CS conceived research, performed statistical analyses, and wrote the manuscript. AGP and PL prepared materials and equipment, performed the VOO analysis, and interpreted analytical data. MM-B prepared materials, designed, and interpreted results of the field experiments. JAN-C contributed to writing the manuscript and interpreted results of the field experiments.

## Conflict of Interest Statement

The authors declare that the research was conducted in the absence of any commercial or financial relationships that could be construed as a potential conflict of interest.

## Footnotes

1 http://www.metaboanalyst.ca
