# Supplementary material for: Insights Into the Effect of Verticillium dahliae Defoliating-Pathotype Infection on the Content of Phenolic and Volatile Compounds Related to the Sensory Properties of Virgin Olive Oil
Source: Front Plant Sci. 2019 Mar 5;10:232. doi: 10.3389/fpls.2019.00232 (PMC6413673; doi:10.3389/fpls.2019.00232)
Supplement: Supplementary file 1 [file Data_Sheet_1.pdf]

SUPPLEMENTARY MATERIAL

Supplementary Table 1. ANOVA analysis to determine the effects of inoculation by defoliating *Verticillium dahliae* and soil type on disease development and yield in plants of cultivar 'Picual' grown under field conditions

**Variable**	**Inoculation Treatment**	**Soil Type**	**Inoculation treatment x Soil Type**
Trees with no yield (%)	<0.001	0.009	0.007
Mean yield per tree (g)	0.008	0.275	0.147
Mean Disease Severity (0-4 scale)[a]		0.002	
Death plants (%)[a]		0.017	
Disease Incidence (%)[a]		0.012	

[a] Data from control treatment were not include in the analysis as all plants sowed no symptoms (mean= 0, variance=0) and cannot be included in the ANOVA analyses

Supplementary Table 2. Mean content of the main groups of phenolic and volatile compounds in oils extracted from olive trees grown in three different soils (sandy-loam, clay-loam, and loam) infected (Vd) and non-infected (control) by D-*Verticillium dahliae* and ANOVA analysis to determine the effects of pathogen inoculation and soil type

	Sandy-Loam				Clay-Loam				Loam				ANOVA Analysis		
**Compound** [b]	**Vd inoculated** [a]		**Control**		**Vd inoculated**		**Control**		**Vd inoculated**		**Control**		**Inoculation Treatment**	**Soil Type**	**Inoculation treatment x Soil Type**
**Phenols (μg/g)**															
**3,4-DHPEA**	1.17	AB	1.23	AB	1.11	B	1.20	B	1.57	A	1.29	A	0.607	**0.027**	0.156
**p-HPEA**	2.87		3.77		3.31		3.06		3.62		4.01		0.175	0.121	0.193
**3,4-DHPEA acetate**	0.63	B*	0.28	B	1.26	A*	0.46	AB	0.77	AB*	0.55	A	**<0.001**	**0.016**	0.084
**3,4-DHPEA-EDA**	15.61		17.96		16.86		16.14		14.29		15.79		0.495	0.604	0.695
**p-HPEA-EDA**	8.21		10.60	*	10.46		11.89	*	8.29		14.14	*	**0.009**	0.341	0.263
**3,4-DHPEA-EA**	187.88		220.37	*	154.56		220.27	*	162.13		180.20	*	**0.018**	0.234	0.440
**p-HPEA-EA**	95.63		131.54	*	89.82		126.32	*	94.49		101.17	*	**0.002**	0.257	0.216
**pinoresinol**	1.87		1.97		2.15		2.01		2.14		1.90		0.181	0.151	0.116
**1-acetoxypinoresinol**	3.93		3.79		3.59		3.75		3.73		3.35		0.524	0.389	0.511
**luteolin**	11.54		10.70		10.04		11.51		10.98		12.07		0.363	0.619	0.279
**apigenin**	4.63		3.72		3.71		4.68		4.08		4.17		0.800	0.952	**0.002**
**vanillic acid**	0.15		0.18	*	0.14		0.19	*	0.15		0.22	*	**0.013**	0.579	0.728
**p-coumaric acid**	0.50		0.58	*	0.43		0.54	*	0.51		0.63	*	**0.007**	0.172	0.852
**cinnamic acid**	0.03		0.03		0.03		0.03		0.03		0.04		0.219	0.543	0.811
**ferulic acid**	0.11		0.11		0.13		0.11		0.14		0.13		0.334	0.069	0.609
**Orthodiphenols**	216.83		250.54	*	183.83		249.58	*	189.75		209.90	*	**0.025**	0.270	0.525
**Tyrosol derivatives**	311.99		385.76	*	277.38		379.35	*	285.16		317.15	*	**0.007**	0.264	0.478
**Lignans**	5.80		5.76		5.73		5.76		5.87		5.25		0.244	0.577	0.286
**Flavonoids**	16.17		14.42		13.75		16.19		15.06		16.24		0.422	0.769	0.089
**Phenolic acids**	0.79		0.91	*	0.73		0.88	*	0.84		1.02	*	**0.011**	0.181	0.871
**Non-orthodiphenols**	117.93		156.29	*	113.77		152.58	*	117.18		129.76	*	**0.001**	0.350	0.303

	Sandy-Loam				Clay-Loam				Loam				ANOVA Analysis		
**Compound** [b]	**Vd inoculated** [a]		**Control**		**Vd inoculated**		**Control**		**Vd inoculated**		**Control**		**Inoculation Treatment**	**Soil Type**	**Inoculation treatment x Soil Type**
**Total phenols**	334.76		406.84	*	297.60		402.17	*	306.93		339.66	*	**0.007**	0.278	0.473
**Volatiles compounds (ng/g)**															
**6C-1**	345.82		455.94	*	281.62		540.49	*	310.80		423.24	*	**0.002**	0.719	0.336
**6C-2**	1369.27	B	3812.97	*	1119.03	C	2712.30	*	1587.76	A	1704.47		**<0.001**	**0.030**	**0.008**
**6C-3**	692.54		1005.80	*	588.81		1020.76	*	735.03		902.40	*	**0.001**	0.903	0.429
**6C-4**	15308.23	*	9666.24		15835.86	*	11789.79		14083.86	*	12103.92		**0.001**	0.587	0.368
**6C-5**	14.67		18.19		14.68		16.91		18.16		42.79		0.247	0.302	0.496
**6C-6**	1247.36		1091.88		1082.14		1331.00		1215.01		1102.68		0.955	0.934	0.278
**6C-7**	172.59		339.54		177.46		205.41		253.72		192.53		0.103	0.155	**0.006**
**6C-8**	665.12		825.15		489.42		682.42	*	593.39		789.76	*	**0.002**	0.053	0.950
**6C-9**	309.33		172.71		264.06		291.32		355.20		262.97		0.064	0.293	0.157
**5C-1**	540.66	A	1148.46	A*	334.69	B	678.27	B*	466.70	AB	810.51	B*	**<0.001**	**<0.001**	**0.032**
**5C-2**	21.15	AB	76.71	*	13.62	B	37.04	*	23.21	A	41.07	*	**<0.001**	**<0.001**	**<0.001**
**5C-3**	65.87	A	108.78	A*	50.51	B	75.86	B*	54.35	B	79.50	B*	**<0.001**	**<0.001**	0.108
**5C-4**	204.69		360.81		134.20		242.31		342.82		256.49		0.147	**0.065**	**0.046**
**5C-5**	487.92		688.88	*	389.95		570.96	*	527.93		657.95	*	**0.001**	0.111	0.821
**5C-6**	37.48	A	49.49	A*	27.72	B	42.95	B*	35.89	A	50.29	A*	**<0.001**	**0.008**	0.821
**5C-7**	225.12		310.27	*	114.61		296.39	*	174.44		295.80	*	**<0.001**	0.124	0.265
**5C-8**	211.73		289.45	*	106.32		265.73	*	187.01		255.41	*	**<0.001**	0.067	0.185
**5C-9**	1037.40		1307.50	*	562.93		1259.04	*	740.36		1216.52	*	**<0.001**	0.051	0.146
**5C-10**	1032.38	A	1457.11	A*	442.64	B	1267.62	B*	833.99	AB	1343.23	AB*	**<0.001**	**0.017**	0.267
**5C-11**	0.00		0.00		0.00		0.00		75.16		0.00		0.327	0.383	0.383
**5C-12**	894.42	A	1145.41	A*	506.96	B	917.85	B*	836.44	AB	1041.46	AB*	**0.002**	**0.019**	0.591
**5C-13**	941.10	A	1207.30	A*	387.37	B	1176.42	B*	679.68	AB	1327.09	AB*	**<0.001**	**0.032**	0.062
**5C-14**	125.05	A	130.18	A	85.15	B	104.59	B	98.66	AB	108.90	AB	0.206	**0.018**	0.804

Compound [b]	Sandy-Loam Vd inoculated [a]		Sandy-Loam Control		Clay-Loam Vd inoculated		Clay-Loam Control		Loam Vd inoculated		Loam Control		ANOVA Analysis Inoculation Treatment	ANOVA Analysis Soil Type	ANOVA Analysis Inoculation treatment x Soil Type
**5C-15**	12.98		11.50		11.62		19.83		12.25		11.00		0.377	0.224	0.104
**5C-16**	6.40		7.95		9.09		7.72		13.64		7.04		0.317	0.473	0.293
**E-1**	373.38	*	115.61		395.53	*	259.17		406.40	*	227.90		**0.001**	0.313	0.574
**E-2**	40.79	B*	14.38	B	42.20	AB*	38.66	A	61.11	A*	34.45	AB	**0.001**	**0.009**	0.109
**E-3**	907.59		641.48		635.70		1231.49		800.79		735.12		0.503	0.507	**0.031**
**E-4**	15.41		14.15		14.96		11.36		12.26		20.70		0.504	0.324	**0.024**
**E-5**	64.89	*	7.92		357.70	*	13.13		761.63	*	23.73		**0.029**	0.225	0.255
**E-6**	47.78		38.41		38.80		53.15		100.29		41.16		0.218	0.241	0.122
**E-7**	13.59	*	10.38		14.79	*	13.81		25.47	*	12.11		**0.015**	0.059	0.075
**BC-1**	36.14		22.36		23.20		28.81		31.16		19.42		0.131	0.723	0.145
**BC-2**	30.95		28.24		16.56		20.44		28.21		27.30		0.987	0.174	0.858
**BC-3**	52.16		22.31		57.21		52.61		53.88		42.78		0.132	0.341	0.554
**T-1 (limonene)**	43.32		77.80	*	25.62		57.54	*	53.22		75.84	*	**0.001**	0.062	0.818
**C6/LnA**	19150.48	*	16390.55		19099.61	*	17616.67		18204.34	*	16472.02		**0.027**	0.804	0.908
**C6/LA**	974.45		997.87	*	753.48		973.74	*	948.59		1052.74	*	**0.031**	0.072	0.299
**C5/LnA**	5699.94	A	8150.17	A*	3071.51	B	6830.42	B*	4978.00	AB	7375.33	AB*	**<0.001**	**0.002**	0.320
**C5/LA**	144.43		149.63		105.87		132.15		124.55		126.94		0.239	0.058	0.540
**Esters**	1463.43	*	842.33		1499.68		1620.79		2167.93	*	1095.17		**0.018**	0.146	**0.048**
**BC**	119.24		72.90		96.97		101.86		113.25		89.49		0.166	0.959	0.405
**Terpenes**	43.32		77.80	*	25.62		57.54	*	53.22		75.84	*	**0.001**	0.060	0.822
**Total volatiles**	27595.28		26681.24		24652.74		27333.17		26589.88		26287.53		0.763	0.842	0.623

[a] Means in a row followed by different letters indicate significant differences ($P < 0.05$) among soil types within each inoculation treatment, according to Tukey HSD test. Means followed by an asterisk indicate that are significantly higher ($P < 0.05$) than those in the other inoculation treatment within each soil type.
[b] Name of compounds is shown in Table 1.

Supplementary Figure 1. Hierarchical cluster analysis and heatmap of quantified phenolic compounds (**A**) and volatile compounds (**B**) in the oils extracted from olive trees grown in three different soils (L: loam; CL: clay-loam; and, SL: sandy-loam) infected (Vd) and non-infected (control) by D-*Verticillium dahliae*. Numbers correspond to the VOO extracted from trees grown in each of five blocks in the experimental plot.

A

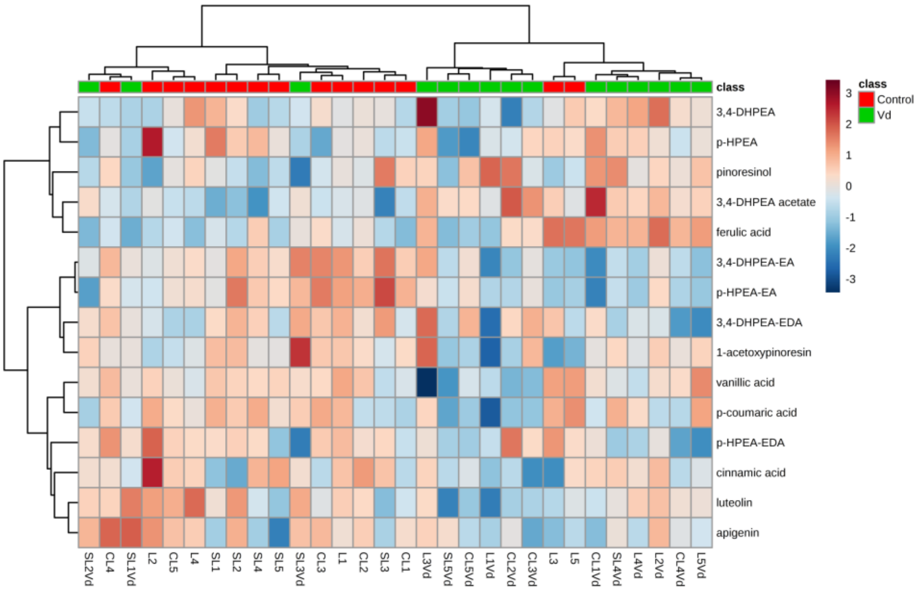

B

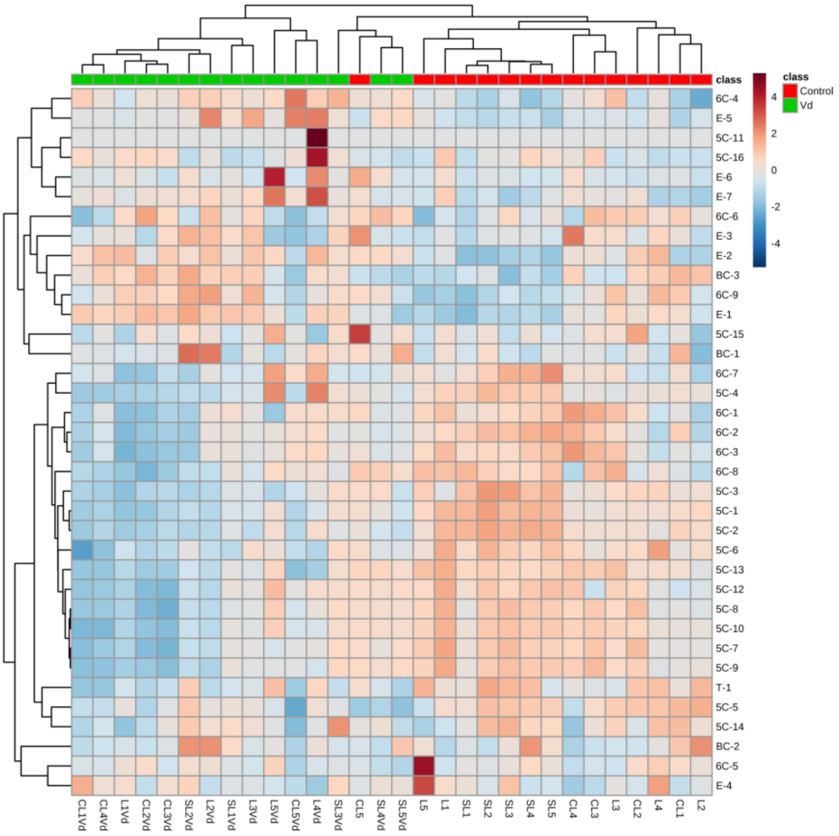

Supplementary Table 3. Mean odor active values (OAV) of the main volatile compounds in oils extracted from olive trees grown in three different soils (sandy-loam, clay-loam, and loam) infected (Vd) and non-infected (control) by D-*Verticillium dahliae*.

			Sandy-Loam		Clay-Loam		Loam	
**Compound**	**Code**	**Descriptor**	**Vd inoculated**	**Control**	**Vd inoculated**	**Control**	**Vd inoculated**	**Control**
(E)-hex-3-enal	6C-1	Not defined	0.77	1.01	0.63	1.20	0.69	0.94
(Z)-hex-3-enal	6C-2	Green, leaf-like	8.05	22.43	6.58	15.95	9.34	10.03
(Z)-hex-2-enal	6C-3	Not defined	1.63	2.37	1.39	2.41	1.73	2.13
(E)-hex-2-enal	6C-4	Green, apple-like	36.10	22.80	37.35	27.81	33.22	28.55
(E)-hex-3-enol	6C-5	Green	0.01	0.01	0.01	0.01	0.01	0.03
(Z)-hex-3-enol	6C-6	Green, leaf-like	1.13	0.99	0.98	1.21	1.10	1.00
(E)-hex-2-enol	6C-7	Green grass, leaves	0.03	0.07	0.04	0.04	0.05	0.04
hexanal	6C-8	Green apple, grassy	2.22	2.75	1.63	2.27	1.98	2.63
hexan-1-ol	6C-9	Fruit, undesirable	0.77	0.43	0.66	0.73	0.89	0.66
pent-1-en-3-one	5C-1	Green, pungent	740.63	1573.23	458.47	929.13	639.31	1110.29
(Z)-pent-2-enal	5C-2	Not defined	0.07	0.26	0.05	0.12	0.08	0.14
(E)-pent-2-enal	5C-3	Green, apple	0.22	0.36	0.17	0.25	0.18	0.27
pent-1-en-3-ol	5C-4	Pungent, butter	0.51	0.90	0.34	0.61	0.86	0.64
(Z)-pent-2-en-1-ol	5C-5	Banana	1.95	2.76	1.56	2.28	2.11	2.63
(E)-pent-2-en-1-ol	5C-6	Pungent green fruity	0.15	0.20	0.11	0.17	0.14	0.20
pentene dimer - 1	5C-7	Alcane	0.02	0.02	0.01	0.02	0.01	0.02
pentene dimer - 2	5C-8	Alcane	0.02	0.02	0.01	0.02	0.01	0.02
pentene dimer - 3	5C-9	Alcane	0.08	0.10	0.04	0.09	0.05	0.09
pentene dimer - 4	5C-10	Alcane	0.08	0.11	0.03	0.09	0.06	0.10
pentene dimer - 5	5C-11	Alcane	0.00	0.00	0.00	0.00	0.01	0.00
pentene dimer - 6	5C-12	Alcane	0.07	0.08	0.04	0.07	0.06	0.08
pentene dimer - 7	5C-13	Alcane	0.07	0.09	0.03	0.09	0.05	0.10
pentan-3-one	5C-14	Sweet, fruity	0.02	0.02	0.01	0.01	0.01	0.02
pentanal	5C-15	Woody, bitter, oily	0.05	0.05	0.05	0.08	0.05	0.05
pentan-1-ol	5C-16	Fruity	0.01	0.02	0.02	0.02	0.03	0.01
hexyl acetate	E-1	Green, fruity, sweet	0.36	0.11	0.38	0.25	0.39	0.22
(E)-hex-2-en-1-yl acetate	E-2	Fruity	0.20	0.07	0.21	0.19	0.31	0.17
(Z)-hex-3-en-1-yl acetate	E-3	Green, banana-like	4.54	3.21	3.18	6.16	4.00	3.68
methyl acetate	E-4	Solvent, fruit	0.08	0.07	0.07	0.06	0.06	0.10
ethyl acetate	E-5	Sticky, sweet	0.07	0.01	0.38	0.01	0.81	0.03
methyl hexanoate	E-6	Pineapple	0.09	0.07	0.07	0.10	0.19	0.08
ethyl hexanoate	E-7	Apple peel	0.03	0.02	0.03	0.03	0.05	0.02
2-methyl-butanal	BC-1	Malty	6.69	4.14	4.30	5.33	5.77	3.60
3-methyl-butanal	BC-2	Malty	5.95	5.43	3.19	3.93	5.43	5.25
2-methyl-butan-1-ol	BC-3	Winey, spicy	0.11	0.05	0.12	0.11	0.11	0.09
limonene	T-1	Citruslike, ethereal	0.17	0.31	0.10	0.23	0.21	0.30
